# Impact of low health literacy on patients’ health outcomes: a multicenter cohort study

**DOI:** 10.1186/s12913-022-08527-9

**Published:** 2022-09-12

**Authors:** Rabia Shahid, Muhammad Shoker, Luan Manh Chu, Ryan Frehlick, Heather Ward, Punam Pahwa

**Affiliations:** 1 Department of Medicine, University of Saskatchewan, 103 Hospital Drive, Saskatoon, SK7N OW8 Canada; 2 College of Medicine, University of Saskatchewan, Saskatoon, Canada; 3 Provincial Research Data Services, Alberta Health Services, Edmonton, Canada; 4 Canadian Center for Health and Safety in Agriculture, University of Saskatchewan, Saskatoon, Canada

**Keywords:** Health literacy, Hospital readmission, Emergency department revisit, Patient outcomes, Length of stay

## Abstract

**Background:**

This study aims to assess the health literacy of medical patients admitted to hospitals and examine its correlation with patients’ emergency department visits, hospital readmissions, and durations of hospital stay.

**Methods:**

This prospective cohort study recruited patients admitted to the general internal medicine units at the two urban tertiary care hospitals. Health literacy was measured using the full-length Test of Functional Health Literacy in Adults. Logistic regression analyses were performed to examine the correlation between health literacy and the desired outcomes. The primary outcome of interest of this study was to determine the correlation between health literacy and emergency department revisit within 90 days of discharge. The secondary outcomes of interest were to assess the correlation between health literacy and length of stay and hospital readmission within 90 days of discharge.

**Results:**

We found that 50% had adequate health literacy, 32% had inadequate, and 18% of patients had marginal health literacy. Patients with inadequate health literacy were more likely to revisit the emergency department as compared to patients with adequate health literacy (odds ratio: 3.0; 95% Confidence Interval: 1.3–6.9, *p* = 0.01). In patients with inadequate health literacy, the mean predicted probability of emergency department revisits was 0.22 ± 0.11 if their education level was some high school or less and 0.57 ± 0.18 if they had completed college. No significant correlation was noted between health literacy and duration of hospital stay or readmission.

**Conclusions:**

Only half of the patients admitted to the general internal medicine unit had adequate health literacy. Patients with low health literacy, but high education, had a higher probability of emergency department revisits.

## Background

Health literacy is the ability of an individual to effectively use their reading, writing, verbal, and numerical skills to contribute to their personal healthcare positively [1, 2]. An individual’s health literacy skills are crucial for health-related decisions. Health literacy is described as “personal knowledge and competencies which enable people to access, understand, appraise, and use information and services in ways which promote and maintain good health and wellbeing for themselves and those around them” [3]. It is essential that patients and their families are able to “... obtain, process, and understand [the] basic health information and services needed to make appropriate health decisions to manage their health.” More than 43 million people in the United States have inadequate health literacy, and nearly half of the adult Canadians have literacy skills below a high school level, affecting their ability to function [4–8].

The relationship between eudcation and health literacy is not well understood. Education has been suggested to be a key factor for adequate health literacy and thereby good health [9, 10]. Evidence shows that people with lower education have lower health literacy skills as compared to people with higher education. Nevertheless, health literacy and general literacy are not identical concepts. Inadequate health literacy is not uncommon among patients with a high level of education [10]. General literacy does not provide all the skills required to manage and communicate critical health information and concerns [11, 12]. Evidence supports an incongruity between the average reading ability of patients and their ability to interpret and comprehend health information, as when managing health and complex diseases, patients require more than the ability to read and manipulate numbers [13].

Inadequate health literacy is recognized as a stronger predictor of poor health than age, income, employment status, education level, or race [14]. It has been found that people with inadequate health literacy often have difficulty understanding prescription labels, participating in medical decisions, following medical recommendations, and attending their follow-up appointments [15, 16]. Individuals with inadequate or marginal health literacy often struggle with poor self-care behaviors, receive fewer preventive measures, and have increased all-cause mortality [17–23]. Williams et al. showed that a quarter of the patients visiting the emergency department had inadequate health literacy, and one-third did not understand how many pills should be taken in their prescription [15]. Inadequate health literacy affects the use of health services and impacts patient satisfaction and the physician–patient relationship [24]. Furthermore, inadequate health literacy is one of the key barriers in the delivery of effective healthcare and quality outcomes [25]. Low health literacy is considered a key source of economic inefficiency in the U.S. healthcare system [25, 26]. It is estimated that inadequate health literacy adds additional 106 to $238 billion cost to the health care system representing 7–17% of all personal healthcare expenditures [26].

People with inadequate health literacy may utilize more resources through more frequent use of inpatient and emergency department visits and have higher care costs with poorer health outcomes. A systematic review examined health literacy in emergency departments and found that a substantial portion of emergency patients does not have adequate health literacy [27]. Howard et al. found an increase in emergency department use and higher costs for patients with inadequate health literacy (as compared to those with adequate literacy) after controlling for age, sex, race or ethnicity, income, education, health behaviors, and chronic conditions [28].

Despite the negative implications of low health literacy, physicians are typically unaware of their patients’ health literacy levels and its subsequent effects on their patients’ outcomes [29]. Health literacy is not routinely evaluated or recorded in patients’ medical records and administrative data. However, health literacy can be an important indicator in evaluating patients’ risks of poor outcomes after hospital discharge and improving patient-physician communication [30].

Most of the studies that have assessed outcomes of patients with inadequate health literacy were performed either in the emergency department or in outpatient settings. Therefore, current knowledge of the outcomes of inadequate health literacy in hospitalized patients with multiple comorbid conditions is limited. This study aims to assess the health literacy of patients admitted to a hospital general medical unit and examine its correlation with emergency room visits, readmissions, and duration of hospital stay.

## Methods

### Setting and study participants

This study was approved by the University of Saskatchewan Research Ethics Board (Bio#308). This prospective cohort study was conducted in the two urban tertiary care hospitals in Saskatoon, Canada. Based on the assumption that approximately 50% patients will have inadequate or marginal health literacy and about 10% pateints will loss to follow up we estimated a smple size of 150 patients with an alpha error of 0.05 with a 2-sided *p* value. This sample size was felt to have adequate power for subgroups analysis. Adult patients admitted to the hospitals’ general internal medicine units were enrolled in the study after written informed consent was obtained. Research assistants recruited patients over the period of June–September 2019. Patients who could read, write, speak English, and were 18 years and older were eligible for enrollment in this study. Patients with known diagnoses of dementia were excluded. Participating patients had their visual acuity checked using a pocket Snellen chart to ensure their ability to complete the assessment.

### Data collection instrument

Health literacy was measured using the full-length Test of Functional Health Literacy in Adults (TOFHLA). The TOFHLA was developed and validated as a measure of functional health literacy used by healthcare providers and researchers [31]. This tool measures health literacy on the assumption that more than general literacy is necessary to understand and negotiate healthcare systems adequately. We used the full-length TOFHLA as it provides richer information about the levels of functioning. It is also recommended to use the full-length TOFHLA when health literacy is used as a dependent or independent variable in research [31]. A license was obtained to reproduce the TOFHLA for use in research from Peppercorn Book and Press Inc.

The TOFHLA assesses an individual’s numeracy and reading comprehension. In this context, numeracy is defined as a patient’s ability to understand and act on numerical directions given by healthcare providers or pharmacists, and reading compression is defined as a patient’s ability to read passages using actual materials from healthcare settings. The test takes 10–20 minutes to complete. The TOFHLA assigns scores into three groups of health literacy: adequate, marginal, and inadequate. An adequate score ranges from 75 to 100 and indicates that patients should be able to read, understand, and interpret most health care texts; a marginal score ranges from 60 to 74 and indicates that patients will have difficulty reading and interpreting health texts; an inadequate score ranges from 0 to 59 and indicates that patients will have difficulty reading, understanding, and interpreting most health materials.

The primary outcome of interest of this study was to determine the correlation between health literacy and emergency department revisit within 90 days of discharge. The secondary outcomes of interest were to assess the correlation between health literacy and length of stay and hospital readmission within 90 days of discharge. Hospital readmission was defined as any admission, for any cause, to either of the two study hospitals within 90 days after discharge from an index hospitalization. If a patient had a subsequent admission after their emergency department revisit, that emergency department revisit was not included in the analysis. We included measures of whether a patient had readmission (yes or no) and the total number of readmissions experienced within 90 days post-discharge. We categorized readmissions into no readmission and ≥ 1 readmission. Similarly, we categorized emergency department revisit within 90 days after discharge into no revisit and ≥ 1 revisit. Length of stay was kept as a continuous variable in days. All eligible patients close to their discharge from a medical unit were provided with the full-length TOFHLA. Patients were prospectively followed, and the length of their hospital stay was recorded. Data for hospital readmissions and emergency department revisits was obtained from the Discharge Abstract Database (DAD) and National Ambulatory Care Reporting System (NACRS).

The following covariates were examined: age groups (< 65 and ≥ 65 years old); sex (male or female); employment status (disabled or injured, retired, and other); household income, marital status; education (some high school or less, completed high school, some college, and complete college); and Charleston Comorbidity Index (CCI).

### Statistical analysis

Data were analyzed using SPSS (Version 26.0, IBM, Armonk, NY, USA) and Stata 13 (Stata Corp, College Station, TX, USA). Descriptive data were presented for patient demographics, health literacy levels’ hospital readmissions, emergency department revisits, and length of stay, including medians with interquartile range values, frequencies, and proportions where applicable. Multivariate logistic regression was performed to examine whether health literacy affects a patient’s emergency department revisits and hospital readmissions, controlling for other possible confounders. Multiple linear regression modeling was conducted after log-transforming to examine the length of stay variable.

Bivariate analyses were conducted first, retaining variables significant at the 0.2 alpha level when modeled alone and with an alpha less than 0.05 in the final multivariate models. In the multivariate levels, interactions among independent variables and covariates were examined. If interaction effects were present, the mean predicted probabilities with their standard deviation were calculated. A two-sided *p* <  0.05 was considered to be significant.

#### Patient and public involvement

Patients were not involved.

## Results

**Fig. 1:**
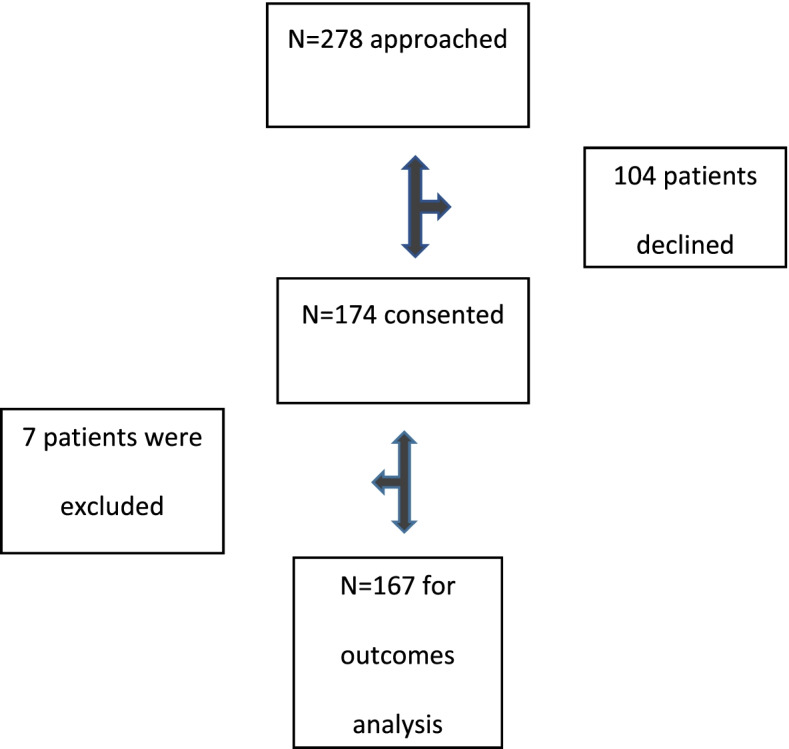
Flow chart showing eligible patients

A total 174 (63%) of 278 eligible patients were consented to be interviewed (Fig. 1). Patient’s characteristics are described in Table 1. In multivariate analysis 7 (4%) patients were excluded due to missing data on length of stay, ER revisits and hospital readmissions. Overall, 46% were men and 54% were women (ratio men/women = 1:1.2). Out of the study population, only 50% of patients had adequate health literacy, 32 and 18% of patients had inadequate and marginal health literacy, respectively. Of the men, 30.4% had inadequate health literacy, 19.0% had marginal health literacy, and 50.6% had adequate health literacy; however, of the women, 33.0% had inadequate health literacy, 17.0% had marginal health literacy, and 50% had adequate health literacy.

**Table 1:** Baseline characteristics of study participants admitted in two urban tertiary care hospitals’ medical units

Patients Characteristics	n (%) 174	Health Literacy			***P*** **value**
		Inadequate	Marginal	Adequate	
		56 (32.0)	31 (18.0)	87 (50.0)	
		%	%	%	
Age Group (in years)					< 0.05
Under 65	92 (53)	20.0	14.4	65.6	
65 and over	82 (47)	45.6	22.8	31.7	
Sex^a^					> 0.05
Male	79 (46)	30.4	19.0	50.6	
Female	94 (54)	33.0	17.0	50.0	
Employment Status					< 0.05
Disabled	35 (20)	34.3	17.1	48.6	
Retired	84 (48)	42.9	20.2	36.9	
Full time	41 (24)	9.8	17.1	73.2	
Other	14 (8)	28.6	7.1	64.3	
Household Income					< 0.05
No income- $20,000	37 (21)	46.0	18.9	35.1	
$20,000-$40,000	36 (21)	30.6	27.8	41.7	
$40,000- $74,999	25 (14)	24.0	16.0	60.0	
$75,000+	36 (21)	16.7	8.3	75.0	
Not stated	40 (23)	40.0	17.5	42.5	
Marital Status					=0.05
Single/Never married	40 (23)	27.5	22.5	50.0	
Divorced/Separated or Widowed	58 (33)	39.7	24.1	36.2	
Married	76 (44)	29.0	10.5	60.5	
Education^b^					< 0.05
Some high school or less	64 (37)	56.3	18.7	25.0	
Complete high school	33 (19)	30.3	15.2	54.6	
Some college	22 (13)	9.1	27.3	63.6	
Complete college	54 (31)	13.0	14.8	72.2	
Charlson Comorbidity Index (CCI) (mean, SD)	3.10 (2.7)	4.7 (2.7)	3.3 (2.2)	2.0 (2.2)	> 0.05
Charlson Comorbidity Index (CCI) (median, IQR)	3.00 (4.0)	4.0 (3.0)	3.0 (3.0)	2.0 (3.0)	> 0.05

^a^information was missing in 1 patient;^b^ information was missing in 1 paitent

Older patients had a significantly higher rate of inadequate health literacy compared to younger patients. In patients over 65 years of age, 45.6% had inadequate health literacy, 22.8% had marginal health literacy, and 31.7% had adequate health literacy; however, in patients under 65 years of age, 20% had inadequate health literacy, 14.4% had marginal health literacy, and 65.6% had adequate health literacy (*p* <  0.05).

Only 24% of patients had full-time employment. Overall, 26.8% of patients who were full-time employed had inadequate or low health literacy as compared with 51.4% of patients who were disabled (< 0.5). Likewise, 64.9% of patients with no income or annual income below $20,000 had inadequate or low health literacy compared with 25% of patients with an annual income of $75,000 or greater (< 0.05). Of all patients, 37% received some high school or less education, 19% completed high school, 13% received some college education, and 31% completed college. In patients who received some high school or less education, 56.3% had inadequate health literacy, 18.8% had marginal health literacy, and 18.4% had adequate health literacy (*p* < 0.05). In patients who completed a college education, 13.0% had inadequate health literacy, 14.8% had marginal health literacy, and 72.2% had adequate health literacy (*p* < 0.05).

Overall, 23% of the patients revisited the emergency department, and 30% required readmission to the hospital at least once within 90 days after their discharge (Table 2). Characteristics of patients who revisited the emergency department are described in Table 3.

**Table 2:** Outcomes of interest according to health literacy of the study participants

Outcomes of Interest	N (%) 167	Inadequate %	Marginal %	Adequate %	***P*** value
ER Revisit					< 0.05
No revisit	129 (77)	27.9	16.3	55.8	
1 or more than re-visit	38 (23)	47.4	21.1	31.6	
Hospital Readmission					> 0.05
No readmission	117 (70)	29.1	15.4	55.6	
1 or more readmission	50 (30)	40.0	22.0	38.0	
Length of Stay in Days (mean, SD)	17.45 (23.01)	19.63 (21.32)	20.24 (27.95)	15.03 (22.20)	> 0.05
Length of Stay in Days (median, IQR)	9.00 (15.00)	12.50 (17.50)	13.00 (18.00)	7.5 (12.00)	> 0.05

*ER* Emergency Room, *SD* Standard Deviation, *IQR* Interquartile Range

**Table 3:** Characteristics of patients who revisited the emergency department

Baseline Characteristics		Emergency Department Revisit		***P*** value
	N (%)	None (%)	More than once (%)	
Health Literacy Score				0.02
Inadequate	54 (32.3)	66.7	33.3	
Marginal	29 (17.3)	72.4	27.6	
Adequate	84 (50.3)	85.7	14.3	
Age Group (in years)				0.54
Under 65	87 (52.5)	77.7	22.4	
65 and over	80 (47.5)	76.6	23.4	
Sex				0.89
Male	77 (46.4)	76.6	23.4	
Female	90 (53.6)	77.5	22.5	
Education				0.21
Some high school or less	61 (36.8)	77.1	23.0	
Complete high school	33 (19.9)	66.7	33.3	
Some college	22 (13.3)	72.7	27.3	
Complete college	50 (30.1)	86.0	14.0	

In univariate analysis, patients who had inadequate and marginal health literacy were more likely to revisit the emergency department as compared to patients with adequate health literacy; the odds ratio (OR) for inadequate health literacy was 3.0 (95% Confidence Interval [CI]: 1.3–6.89, *p* = 0.01), whereas the OR for emergency department revisit for marginal health literacy was 2.28 (95% CI: 0.82–6.32, *p* = 0.11) (Table 4).

**Table 4:** Univariate logistic regression results for health literacy levels, emergency department revisits, and hospital readmissions’ association

		Emergency Department Revisit OR_**unadj**_ (95% CI)^**b**^	*P* value	Hospital Readmission OR_**unadj**_ (95% CI)^**b**^	***P*** value
**Total health literacy score**^**a**^	**Inadequate**	3.00 (1.30–6.90)	0.01	2.01 (0.95–4.27)	0.069
	**Marginal**	2.28 (0.82–6.32)	0.11	2.01 (0.84–5.18)	0.111

^a^ Reference: Adequate, ^b^ *OR*_*unadj. *_Unadjusted Odds Ratio, *CI* Confidence Interval

The final model for multivariate analysis was adjusted for education, age, sex, CCI, and marital status. In multivariate analysis, the test for interaction between health literacy and education status was positive. Therefore, mean predicted probabilities and standard deviations were calculated on the interaction between health literacy and education status.

In patients with inadequate health literacy, the mean predicted probability (±SD) of emergency department revisit was 0.22 ± 0.11 if they had received some high school or less education and 0.57 ± 0.18 if they had completed a college (Table 5). In other words, in patients with inadequate health literacy, two of ten with no college education and six of ten with a college education, revisited the emergency department within 90 days of their discharge. In patients with marginal health literacy, the mean predicted probability (±SD) of revisiting the emergency department was 0.25 ± 0.08 if they had completed some high school or less education and 0.15 ± 0.08 if they had completed a college. In patients with adequate health literacy, the mean predicted probability (±SD) of revisiting the emergency department was 0.24 ± 0.12 if they had received some high school and or less education and 0.10 ± 0.04 if they had completed college.

**Table 5:** Mean predicted probabilities for emergency department revisit stratified by education levels and health literacy scores^a^

Health Literacy	Some High School or Less Mean (SD)	Complete High School Mean (SD)	Some College Mean (SD)	Complete College Mean (SD)
Inadequate (0–59 points)	0.22 (0.11)	0.50 (0.19)	0.50 (0.10)	0.57 (0.18)
Marginal (60–74 points)	0.25 (0.08)	0.4 (0.19)	0.33 (0.21)	0.15 (0.08)
Adequate (75+ points)	0.24 (0.12)	0.22 (0.11)	0.23 (0.11)	0.05 (0.04)

*SD* Standard Deviation, ^a^Adjusted For Age, Sex, Education, Marital Status, And Comorbidity (Cci)

In the univariate analysis of all patients who had at least one hospital readmission, 40% had marginal health literacy, 22% had inadequate health literacy, and 38% had adequate health literacy; however, this was not statistically significant (*p* = 0.115).

The median duration of hospital stay was 9 days. Patients with inadequate health literacy had a median duration of hospital stay of 12.5 days, while patients with marginal and adequate health literacy had median durations of 13 days and 7.5 days (*p* > 0.05), respectively. Regarding the length of stay, in univariate regression analysis, the average duration was higher for patients with marginal and inadequate health literacy; however, this was not statistically significant.

## Discussion

Our findings indicate a low rate of adequate health literacy in hospitalized patients in medical units. Only about 50% of hospitalized patients had adequate health literacy, suggesting that almost half of the hospitalized patients in medical units have difficulty reading, understanding, and interpreting healthcare information. Patients with inadequate health literacy were more likely to revisit the emergency department as compared to patients with adequate health literacy. Patients with low health literacy, but high education, had a higher probability of emergency department revisits. No significant correlation was noted between health literacy and duration of hospital stay or readmission**.**

To the best of our knowledge, there is a scarcity of research on health literacy, and its impact on patients’ emergency department revisits and hospital readmissions after their discharge from the medical units in hospitals. The reported prevalence of inadequate health literacy in hospital settings ranges from 29 to 76.7% in the literature [32–34]. This wide range can be due to the use of different health literacy tools in diverse patient populations. There was no difference in health literacy scores between men and women in this study which is consistent with the literature [35]. Our findings indicate that a higher number of patients older than 65 years of age had inadequate or marginal health literacy as compared to younger patients, which is also found in previous research [34, 36]. The lower level of health literacy in the older population could be due to the decline in comprehension, memory, and word recognition abilities that occur in older age. Although patients with a known diagnosis of dementia were excluded [37], some may have had undetected mild cognitive deficits, which influenced this study’s results. In addition, social determinants of health, including low or no income or disability, were correlated with a lower level of health literacy.

We found that patients with low health literacy scores had a higher probability of emergency department revisits when controlling for other factors, including age, sex, marital status, comorbidity, and education level. Notably, we observed that patients with low health literacy and low education levels had a significantly lower probability of revisiting the emergency department compared to patients with low health literacy and high education levels. It has been previously shown that low education was associated with a higher probability of emergency department visits after surgery [38]. The data data in patients who were admitted to general internal medicine is sparse. A retrospective secondary analysis of clinical trial dataset in medical patients examined the relationship between health literacy and hospital reutilization within 30 days of discharge [32]. The study showed that 49% patients had inadequate or marginal health literacy and that inadequate health literacy was an independent factor for 30-day hospital reutilization after discharge.

Given our findings, it is plausible that not only education but education and health literacy combined have an essential role in the probability of patients revisiting the emergency department and thus their health outcomes. To our knowledge, this study is the first to report that patients with low health literacy but high education have a higher probability of revisiting the emergency department than patients with low health literacy and low education. Further data is needed to identify the critical factors in this result. It is plausible that patients with low education and low health literacy are not fully aware of the harmful effects of underlying illness and are, therefore, less likely to seek early medical attention. Patients with low health literacy may not fully understand and follow the medical instructions provided to them at their hospital discharge, and the consequent inability to attend follow-up visits and delays in seeking medical attention could result in detrimental effects on their health outcomes.

This study has many implications and contributions to society and the medical field at large. Our findings underscore the importance of health literacy interventions. Health education can reduce a patient’s probability of revisiting the emergency department. It is vital to develop and evaluate interventions that run from during a patient’s stay in hospital to their discharge that aim to improve health knowledge. Similarly, it would be necessary to explore factors outside the hospital setting that decreases emergency department revisits for patients with low health literacy. These factors could include access to primary care physicians, support in the community, and knowledge of underlying medical illnesses [39].

Unlike earlier reports [40–43], this study did not find a significant association between low health literacy and duration of stay or hospital readmission. Patients with inadequate or marginal health literacy had an average length of stay almost twice that of patients with adequate health literacy; however, this was not statistically significant in univariate analysis.

The key strengths of our study are that patients were followed prospectively, and a validated tool was used to assess health literacy. Furthermore, we were able to adjust for essential variables in multivariate modeling. However, our study has some limitations; significant limitations are that non-English speaking patients were not included, and the sample size was relatively small. We were not able to capture if patients had readmission or revisits to the ER for the same or differrent medical conditions due to multiple medical conditons. In addition, instead of short TOFHLA we used full-lenght TOFHLA which takes about 20 minutes to complete. A comprehensive measurement of health litrecacy in the acute care setting may be challenging for some patients as refelcted by 37% refusal rate to particpate in our study. Lastly, our patient population was sampled from two tertiary care hospitals, and although both hospitals received referrals from remote communities, most of the patients were likely from urban areas; therefore, rural and remote populations were underrepresented in this sample, making the results not generalizable to the greater population.

## Conclusions

Our results indicate that only half of the patients admitted to general internal medicine units have adequate health literacy. Patients with inadequate or marginal health literacy scores are more likely to revisit the emergency room within 90 days following their discharge from the hospital. It is prudent to address the needs of the population with marginal and inadequate health literacy as improved health literacy can have positive effects on healthcare systems and individuals’ health outcomes. Future studies are required to identify and address strategies for improving the health outcomes of people with inadequate health literacy.

## Data Availability

The datasets generated and/or analysed during the current study are not publicly available due the confidentiality of the participants but are available from the corresponding author on reasonable request.
